# Exploring the Association Between Psychological Inflexibility, Experiential Avoidance and Sleep-Related Problems Among Adolescents: The EHDLA Study

**DOI:** 10.3390/healthcare14142183

**Published:** 2026-07-20

**Authors:** María Paula Zalamea-Delgado, Estela Jiménez-López, Arthur Eumann Mesas, Rodrigo Yáñez-Sepúlveda, Héctor Gutiérrez-Espinoza, Jorge Olivares-Arancibia, Yasmin Ezzatvar, Brendon Stubbs, Lee Smith, Lucille Ridgell, Camila Miño, José Francisco López-Gil

**Affiliations:** 1 School of Medicine, Universidad Espíritu Santo, Samborondón, Guayaquil 0901952, Ecuador; 2 Health and Social Research Center, Universidad de Castilla-La Mancha, 16002 Cuenca, Spain; 3 Centro de Investigación Biomédica en Red de Salud Mental, Instituto de Salud Carlos III, 28029 Madrid, Spain; 4 Faculty Education and Social Sciences, Universidad Andres Bello, Viña del Mar 2520000, Chile; 5 Faculty of Education, Universidad Autónoma de Chile, Santiago 7500138, Chile; 6 AFySE Group, Research in Physical Activity and School Health, School of Physical Education, Faculty of Education, Universidad de las Américas, Santiago 8320000, Chile; 7 Lifestyle Factors with Impact on Ageing and Overall Health (LAH) Research Group, Department of Nursing, University of Valencia, 46010 Valencia, Spain; 8 Vicerrectoría de Investigación y Postgrado, Universidad de Los Lagos, Osorno 5290000, Chile; 9 Department of Psychological Medicine, Institute of Psychiatry, Psychology and Neuroscience (IoPPN), King’s College London, London WC2R 2LS, UK; 10 Center for Sport Science and University Sports, University of Vienna, 1010 Wien, Austria; 11 Centre for Health Performance and Wellbeing, Anglia Ruskin University, Cambridge CB1 1PT, UK; 12 Department of Public Health, Faculty of Medicine, Biruni University, Istanbul 34015, Turkey; 13 Carver University, Orlando, FL 32804, USA; 14 Universidad Internacional para el Desarrollo (UNINDE), Extremadura, 06080 Badajoz, Spain; 15 Department of Sport Sciences, Faculty of Sport and Health Sciences, Fit Generation Research Institute, AD500 Andorra la Vella, Andorra

**Keywords:** sleep disturbances, psychological resiliency, avoidance behaviors, adolescent, cross-sectional studies, psychological inflexibility, experiential avoidance

## Abstract

**Highlights:**

**What are the main findings?**

- In a representative sample of 623 Spanish adolescents, higher psychological inflexibility and experiential avoidance scores (measured by the AAQ-II) were significantly associated with a greater probability of bedtime problems, excessive daytime sleepiness, nocturnal awakenings, and irregular sleep patterns, with each additional AAQ-II point increasing the likelihood of any sleep-related problem by 1.2%.
- The association between psychological inflexibility and sleep difficulties was consistent across most sleep domains assessed by the BEARS questionnaire, with snoring being the only dimension showing no significant relationship.

**What are the implications of the main findings?**

- Higher psychological inflexibility may serve as a screening marker for identifying adolescents at elevated risk of sleep-related problems in clinical and school settings.
- Future longitudinal and intervention studies (particularly those testing acceptance and commitment therapy-based approaches) are needed to establish the directionality of these associations and evaluate whether improving psychological flexibility translates into measurable improvements in adolescent sleep outcomes.

**Abstract:**

**Background**: Adolescence is a sensitive period during which sleep undergoes substantial change, increasing vulnerability to sleep disturbances and their health consequences. Psychological inflexibility (PI) and experiential avoidance (EA), operationalized through the Acceptance and Action Questionnaire-II (AAQ-II), have been associated with poorer sleep among adults; however, evidence in adolescents remains scarce. This study examined the association between PI, EA, and sleep-related problems in a representative sample of Spanish adolescents. **Methods**: A cross-sectional analysis was conducted using data from 623 adolescents (aged 12–17 years; 56.7% girls) from the Eating Healthy and Daily Life Activities (EHDLA) study, conducted in the *Valle de Ricote* (Murcia, Spain) during 2021–2022. PI and EA were assessed with the Spanish-validated AAQ-II; sleep-related problems were screened using the BEARS (B = Bedtime problems, E = Excessive daytime sleepiness, A = Awakenings during the night, R = Regularity and duration of sleep, S = Snoring) questionnaire across five domains. Generalized linear models were adjusted for sex, age, socioeconomic status, physical activity, sedentary behavior, body mass index (BMI) z-score, energy intake, and sleep duration. **Results**: Higher AAQ-II scores were significantly associated with increased probabilities of bedtime problems (+0.9% per point; 95% confidence interval [CI]: 0.6–1.1%), excessive daytime sleepiness (+1.0%; 95% CI: 0.7–1.2%), nocturnal awakenings (+0.7%; 95% CI: 0.5–1.0%), and problems with sleep regularity and duration (+0.8%; 95% CI: 0.5–1.1%). Each additional AAQ-II point increased the probability of reporting any sleep-related problem by 1.2% (95% CI: 0.9–1.5%; *p* < 0.001). Corresponding adjusted odds ratios ranged from 1.04 to 1.06 per point. No significant association was found for snoring. **Conclusions**: In this cross-sectional study, psychological inflexibility and experiential avoidance were positively associated with several sleep-related problems among adolescents. Longitudinal and intervention studies are warranted before causal or clinical recommendations can be made.

## 1. Introduction

Adolescence represents a transition period between childhood and adult life [1], during which sleep often experiences profound changes [2]. Young individuals tend to sleep later at night, adopt a late chronotype [3], prefer evening activities [2,4], and show decreased total sleep time during schooldays [4]. Consequently, adolescents are prone to sleep loss, sleep deprivation [2], and sleep-related problems (such as difficulty falling asleep, excessive daytime sleepiness, awakenings during the night, irregular sleep patterns and durations, as well as snoring) [5]. A review of secular trends among children and adolescents has confirmed a consistent and rapid decline in sleep duration [6], which negatively affects sleep quality, resulting in non-restorative sleep, difficulty initiating sleep and difficulty maintaining sleep [7,8]. Common reasons why insufficient sleep occurs in this age group include television watching, internet usage, gaming, entertainment [4,9] and early school start times [4,10]. Given that sleep disturbances are associated with an increased risk of developing psychiatric disorders, including depression, drug use, and nicotine dependence [11], this has become an important topic of public health research into adolescent health.

Studies have shown that psychological inflexibility (PI), which manifests as a tendency to rely on automatic emotional reactions rather than value- and goal-driven actions [12], is also related to sleep disturbances [13,14]. PI arises from six interrelated processes, one of which is experiential avoidance (EA) [15]. EA characterizes a persistent behavioral pattern in which an individual attempts to suppress, circumvent, or modify unwanted internal states (such as distressing cognitions, emotions, or bodily sensations) as well as the situational contexts that provoke them [16]. When these avoidance strategies dominate responding, behavior becomes governed by self-critical judgments rather than by values and goals. The cumulative effect is a narrowing of present-moment awareness and a drift away from meaningful, long-term pursuits [15]. These constructs are operationalized using the Acceptance and Action Questionnaire-II (AAQ-II), a seven-item self-report measure with well-established convergent and predictive validity [12].

Although previous studies have investigated the association between PI and sleep disturbances in adults, the scientific literature concerning adolescents is scarce. A meta-analysis found that psychological flexibility and experiential acceptance (identified with lower AAQ-II scores) are positively associated with better psychological outcomes and higher quality of life measures [15]. Other studies have supported this direction of association, highlighting PI as a psychological vulnerability factor that may contribute to increased emotional distress and poorer sleep outcomes [17,18,19]. By impairing emotional regulation in children and adolescents [20], PI may contribute to difficulties in falling and staying asleep, among other difficulties [21]. Additionally, it can result in avoidant coping [22] that ultimately disrupts sleep patterns [23]. The combination of these factors creates a cycle in which poor sleep exacerbates stress and emotional difficulties, which further reinforce sleep disturbances [24,25]. PI and EA may influence sleep through cognitive fusion, rumination, and heightened arousal, although anxiety, depression, and circadian factors represent important competing explanations. For example, one study identified two models of association: in one, PI was associated with sleep difficulty and mediated by depressive symptoms; in the other, PI was associated with depressive symptoms and mediated by sleep difficulty [18]. AAQ-II scores were analyzed alongside insomnia severity index scores in adults in another study, where associations were found only between PI and severe insomnia [19].

Clarifying the relationship between PI, EA, and sleep difficulties could inform both clinical screening and public health interventions in adolescent populations. Accordingly, this study examined the association between PI/EA (as measured by the AAQ-II) and a range of sleep-related problems in a community sample of Spanish adolescents. Therefore, the aim of this study was to examine the cross-sectional association between psychological inflexibility and experiential avoidance and sleep-related problems among Spanish adolescents, adjusting for sociodemographic, lifestyle, and anthropometric covariates.

## 2. Materials and Methods

### 2.1. Study Design and Population

Participants with missing AAQ-II data (*n* = 661, 48%) were compared with those included in the analytic sample (Supplementary Table S1). Data were collected between 2021 and 2022, during the Coronavirus Disease 2019 (COVID-19) pandemic, when mobility restrictions and disrupted routines may have affected adolescent sleep and psychological distress [26,27].

The Eating Healthy and Daily Life Activities (EHDLA) project, a cross-sectional study, examined a representative sample of Spanish adolescents in 2021 and 2022. The data were collected from students from three secondary schools in the region, and the protocol has already been published [28]. Among the initial 1378 adolescents (100.0%) in the EHDLA study, 661 (48.0%) were excluded because they did not have data on AAQ-II. Additionally, 94 participants were excluded for not having information on sleep disturbances (19 participants, or 1.4%) or missing data on other factors such as energy intake (40 participants, or 2.9%), body mass index (34 participants, or 2.5%), and physical activity (1 participant, or 0.1%). To minimize the risk of bias introduced by missing data, a complete case analysis was performed, retaining only participants with full information across all variables of interest. The final analytic sample comprised 623 adolescents (56.7% girls). Additionally, to strengthen confidence in the findings, extensive sensitivity analyses are presented in Supplementary Table S2, including models excluding sleep duration as a covariate, models adjusting for Depression, Anxiety and Stress Scale-21 Items (DASS-21) anxiety and depression subscales, AAQ-II tertile analyses, and sex-stratified models.

The inclusion criteria for the EHDLA study were as follows: (1) being aged between 12 and 17 years, (2) registered or living in *Valle de Ricote* (Region of Murcia, Spain), and (3) providing written, informed consent from both the legal guardians and participants. Exclusion from the study occurred if the participants were exempt from physical education at school, had medical conditions that prevented them from participating in physical education or were receiving any pharmacological treatment.

The study was approved by the Bioethics Committee of the University of Murcia (ID 2218/2018, approved on 18 February 2019) and the Ethics Committee of the Albacete University Hospital Complex and the Albacete Integrated Care Management (ID 2021-85, approved on 23 November 2021). In accordance with the Helsinki Declaration, the human rights of all participants were respected.

### 2.2. Study Variables

#### 2.2.1. Psychological Inflexibility (Independent Variable)

In the present sample, internal consistency was excellent for the AAQ-II (Cronbach’s α = 0.93). BEARS (B = Bedtime problems, E = Excessive daytime sleepiness, A = Awakenings during the night, R = Regularity and duration of sleep, S = Snoring) items were analyzed as separate screening domains rather than a unidimensional scale (Cronbach’s α = 0.54), consistent with its design as a multi-domain pediatric sleep screen.

To assess PI and EA, the AAQ-II [12] was used. This tool consists of 7 items on a 7-point Likert scale [29]. The total score ranges from 7 to 49, with lower scores indicating low levels of PI and EA and higher scores reflecting elevated levels of both [29]. In the assessment, questions were designed to evaluate two aspects: aversion to undesired feelings and thoughts and the ability to adapt behaviors to achieve long-term goals. To assess aversion, participants were asked items that capture fear of one’s own emotional states and the perceived inability to regulate worry and distress. Items addressing behavioral adaptability examine whether intrusive memories and persistent concerns interfere with goal-directed functioning and the ability to lead a personally meaningful life. The AAQ-II has been translated into Spanish and proven to be reliable and valid in the assessment of PI and EA [29].

#### 2.2.2. Sleep-Related Problems (Dependent Variable)

To evaluate sleep history and screen for the most common sleep disturbances, the BEARS questionnaire was used [30]. This age-appropriate clinical screening tool covers five domains that are particularly relevant across the developmental spectrum: difficulty at bedtime, excessive daytime sleepiness, nocturnal awakenings, problems with sleep regularity and duration, and snoring. As the study focused on an adolescent population, participants completed the questionnaire themselves [28]. In addition to screening for these issues, the BEARS includes a component addressing the duration of sleep, measured as a continuous variable in minutes, allowing for a more detailed analysis of overall sleep patterns. Each BEARS item is answered dichotomously (yes/no answer), with higher frequencies of affirmative responses indicating greater sleep-related problems. While the questionnaire does not yield a composite score, the individual dimensions are analyzed independently to identify specific patterns of disturbance. The screening tool has been translated into Spanish and has demonstrated satisfactory concurrent validity [30].

#### 2.2.3. Covariates

Information such as sex and age was self-reported. Socioeconomic status was examined with the Family Affluence Scale (FAS-III) [31]. This six-item tool evaluates parameters such as car ownership, whether the adolescent has a personal bedroom, the number of computers in the house, the number of bathrooms, the number of people owning a dishwasher, and the number of out-of-the-country holidays taken in the past year. Higher scores denote greater socioeconomic status, ranging from 0 to 13 points. To record physical activity and sedentary behavior, the Youth Activity Profile questionnaire (YAP-S) was used [32]. The tool consists of a 7-day recall by the examined population concerning (1) activity at school, (2) out-of-school activity, and (3) sedentary habits, all of which are measured using a 5-point Likert scale. Body weight and height are measured in kilograms and meters, respectively, following standardized protocols [28]. The body mass index was subsequently calculated by dividing body weight (in kg) by height (in meters). BMI z-scores were derived from these anthropometric data and used as the adiposity covariate in regression models. Energy intake was examined via a self-reported food frequency questionnaire, which has been validated for the Spanish population [33].

### 2.3. Statistical Analysis

To conduct the statistical analyses, R statistical software (version 4.4.0) from the R Core Team in Vienna, Austria, and RStudio (2024.04.1+748) from Posit in Boston, MA, USA, were used. A *p* value <0.05 was considered statistically significant.

To assess the normality of variables’ distributions, density plots and quantile–quantile (Q–Q) plots were used, and the evaluation was further supported by the Shapiro–Wilk test. Categorical variables are displayed as frequencies (*n*) and percentages (%), whereas medians and interquartile ranges (IQRs) are used to describe continuous variables that have a nonnormal distribution. The data collected from female and male adolescents was analyzed together since no relevant interaction between PI and sex in relation to sleep-related problems (*p* = 0.060) was found.

Generalized linear models (GLMs), including advanced statistical methods that manage heteroscedasticity and outliers, were used to assess associations between individual and overall PI and sleep-related problems in adolescents [34]. When addressing GLMs with a binomial distribution, the “Mqle” (M-estimator based quasi-likelihood estimation) method was employed. Using the adolescents’ PI scores, predictive probabilities (%) for the presence of any sleep-related problem as well as specific sleep-related problems were calculated, along with their corresponding 95% confidence intervals (CIs). Covariates including age, sex, socioeconomic status, energy intake, physical activity, sedentary behavior, BMI z-score, and sleep duration were adjusted in models. Multicollinearity was assessed using variance inflation factors, and a variance inflation factor <5 is considered acceptable. Model calibration was evaluated with Hosmer–Lemeshow tests and area under the receiver-operating characteristic curve (ROC) (Supplementary Tables S3 and S4).

A complete case analysis was applied, including only participants with complete data on all study variables, to reduce the potential for bias introduced by missing covariate or exposure data.

## 3. Results

Table 1 presents the descriptive data of the adolescents who participated in the study. The median AAQ-II score was 22.0 (IQR = 17.0). Regarding sleep-related problems, 58.9% of participants reported at least one. Specifically, 24.1% reported bedtime problems, 33.4% experienced excessive daytime sleepiness, 16.4% reported nocturnal awakenings, 30.3% presented problems with sleep regularity and duration, and 6.1% reported snoring.

**Figure 1:**
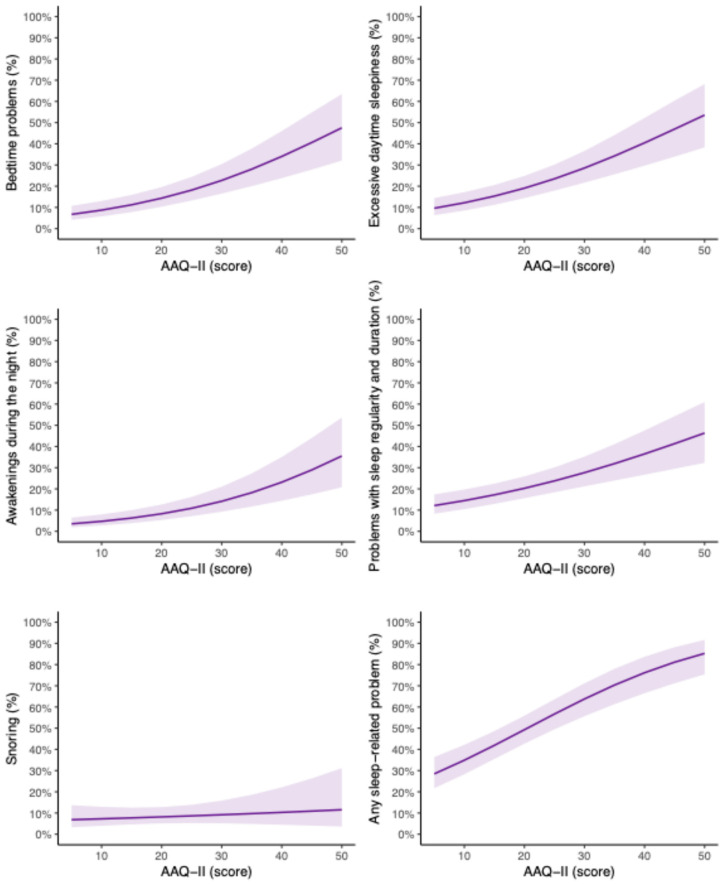
Predictive probabilities of sleep-related problems based on the Acceptance and Action Questionnaire-II (AAQ-II score) among Spanish adolescents. Analyses were adjusted for age, sex, socioeconomic status, energy intake, physical activity, sedentary behavior, body mass index (z-score), and sleep duration. AAQ-II, Acceptance and Action Questionnaire-II.

Figure 1 displays the predictive probabilities of each sleep-related problem across the range of AAQ-II scores. Higher PI was associated with greater probability of sleep-related problems across most domains. For each additional point on the AAQ-II, statistically significant increases were observed in the probability of bedtime problems (+0.9%; 95% CI: 0.6–1.1%; *p* < 0.001), excessive daytime sleepiness (+1.0%; 95% CI: 0.7–1.2%; *p* < 0.001), nocturnal awakenings (+0.7%; 95% CI: 0.5–1.0%; *p* < 0.001), and problems with sleep regularity and duration (+0.8%; 95% CI: 0.5–1.1%; *p* < 0.001). Each additional AAQ-II point also increased the probability of reporting any sleep-related problem by 1.2% (95% CI: 0.9–1.5%; *p* < 0.001). No significant association was found between AAQ-II scores and snoring. In adjusted binomial GLMs, each one-point increase in AAQ-II score was associated with higher odds of bedtime problems (odds ratio [OR] = 1.06, 95% CI: 1.04–1.08), excessive daytime sleepiness (OR = 1.05, 95% CI: 1.04–1.07), nocturnal awakenings (OR = 1.06, 95% CI: 1.04–1.09), problems with sleep regularity and duration (OR = 1.04, 95% CI: 1.02–1.06), and any sleep-related problem (OR = 1.06, 95% CI: 1.04–1.08), but not snoring (OR = 1.01, 95% CI: 0.97–1.05) (Table 2). Although per-point associations were modest, comparing adolescents at the 25th versus 75th percentile of AAQ-II scores corresponded to an absolute increase of 22.9 percentage points in the predicted probability of any sleep-related problem (51.6% to 74.5%). Full model outputs are reported in Tables S5–S10.

## 4. Discussion

The present study investigated the association between PI, EA and sleep-related problems among Spanish adolescents. In the sample, elevated AAQ-II scores were associated with a higher frequency of sleep-related problems across BEARS domains, showing a significant association between PI and sleep-related problems. Specifically, adolescents with higher levels of PI and EA were more likely to experience bedtime problems, excessive daytime sleepiness, awakening during the night, and problems with sleep regularity and duration. A systematic review and meta-analysis examining psychological flexibility–focused interventions, such as acceptance and mindfulness-based therapies, including acceptance and commitment therapy, revealed improvements in individuals with insomnia and sleep disturbances [13,14]. However, the studies included in these articles focused primarily on adults over 18 years of age and who were diagnosed with insomnia, making them relevant but not fully aligned with the present study, which focused on PI, EA, and sleep-related problems in adolescents [13,14].

Furthermore, the present results are consistent with earlier studies in adults, in which PI has been associated with avoidant coping and, correspondingly, with poorer well-being [22] and with reduced sleep efficiency and longer sleep latency [23]. In adults, a previous systematic review and meta-analysis explored associations between six core processes of psychological flexibility and poor sleep outcomes, including subjective sleep quality, sleep latency, sleep duration, habitual sleep efficiency, sleep disturbances, use of sleeping medication, and daytime dysfunction [35]. The review concluded that mindfulness, committed action, acceptance, and diffusion (key psychological flexibility processes) were negatively correlated with poor sleep quality [35].

Although the precise mechanisms by which PI relates to sleep remain unclear, research indicates that psychological flexibility may act as a moderator and a protective factor [36]. It has been reported to buffer the influence of negative predictors–such as stressful life events, daily stress, and low social support–while also being associated with greater well-being and better physical and psychological health [36]. Such flexibility may therefore be associated with fewer sleep-related problems, although this possibility remains to be confirmed in prospective research.

Several interrelated and non-mutually exclusive processes may help account for the observed associations, although all remain hypothetical given the cross-sectional design. One frequently proposed pathway is emotional dysregulation. PI, and specifically cognitive fusion (the tendency to respond to thoughts as literal facts rather than as transient mental events), has been linked to difficulties in modulating negative affect and to maladaptive emotional responding that further compounds EA [15,20,37,38]. Because attempts to suppress or control unwanted internal states tend to increase rather than reduce their frequency and distress [38], the resulting difficulties in processing negative emotions have themselves been associated with sleep disturbances [39,40]. Closely related, PI is consistently associated with anxiety and depressive symptomatology and, more broadly, with general psychological distress and psychopathology, even outside diagnosed disorders [38,41,42], partly through shared features such as rumination and cognitive rigidity [43,44,45]; these symptoms in turn share a well-documented bidirectional relationship with sleep problems such as difficulty maintaining sleep and low daytime energy [46,47]. General distress may therefore partly underlie the association between PI/EA and sleep, an interpretation consistent with the attenuation—though not elimination—of our estimates after adjustment for DASS-21 anxiety and depression.

A further, complementary pathway is heightened arousal. PI has been associated with elevated physiological and cognitive activation, including tension, worry, and hyperarousal, particularly among individuals with anxiety symptoms [13,44,48,49]. Because sleep onset depends on a progressive reduction in physiological and cognitive activation, intrusive sleep-focused cognitions and the emotional charge attached to them may escalate into hyperarousal and anticipatory anxiety; without the flexibility to disengage from these ruminative loops, this arousal could become self-sustaining and perpetuate sleep difficulties [49,50]. Consistent with this account, interventions targeting psychological flexibility to reduce arousal have been proposed and studied as potential treatments for insomnia [13,48,49], although such evidence derives largely from adults and cannot establish directionality in the present cross-sectional sample.

The interpretation of the findings described in the present study should be analyzed in detail due to some relevant limitations. Because this was a cross-sectional study, no direction or causal conclusions can be drawn. A longitudinal study is needed to validate whether higher reported PI and EA lead to a greater incidence of sleep disturbances. Moreover, the direction of association between variables cannot be established. The PI, EA and sleep-related problems data were collected through self-report questionnaires, which allows for the presence of recall and desirability bias, which are common limitations with this type of information recollection. Lastly, given that 48% of the initial sample was excluded due to missing AAQ-II data, there is a potential risk of selection bias. To address this, a complete case analysis was performed, retaining only participants with full data across all study variables. While this approach reduces bias from partially observed records, it assumes that data are missing at random; if missingness in the AAQ-II was systematically related to levels of PI or EA themselves, some residual selection bias cannot be excluded, and findings should be interpreted with appropriate caution. The analyzed sample may differ systematically from those excluded, which could affect the representativeness and external validity of the results. Furthermore, PI, EA, and sleep problems were evaluated via validated approaches, which implement robust data analysis techniques for their examination. Additionally, this study contributes cross-sectional evidence on an association that has been rare before, particularly within this age group. Another major strength is the adjustment for numerous sociodemographic, lifestyle, and anthropometric covariates, which further enhances the reliability of the findings. Data collection during the COVID-19 pandemic may have influenced both sleep patterns and psychological distress among adolescents in Spain, consistent with reports documenting pandemic-related sleep changes in youth [27]. Anxiety and depressive symptoms were not included in primary models because they may lie on the causal pathway between psychological inflexibility and sleep disturbance. In sensitivity analyses adjusting for DASS-21 anxiety and depression subscales, associations were attenuated but remained statistically significant for any sleep-related problem, bedtime problems, nocturnal awakenings, and problems with sleep regularity and duration. Mechanistic pathways involving emotional dysregulation, rumination, and hyperarousal remain hypothetical.

## 5. Conclusions

This cross-sectional study found that PI and EA, measured by the AAQ-II, were positively associated with several adolescent sleep-related problems. Associations persisted after covariate adjustment and were attenuated but largely remained significant in sensitivity analyses. Given the observational/cross-sectional design, directionality and causality cannot be established. Future longitudinal and experimental studies should examine whether interventions targeting psychological flexibility are followed by improvements in adolescent sleep health.

## Figures and Tables

**Table 1:** Descriptive data of the study participants (*n* = 623).

Variable		Total
Age (years)	Median (IQR)	14.0 (2.0)
Sex	Boys (%)	270 (43.3)
	Girls (%)	353 (56.7)
FAS-III (score)	Median (IQR)	8.0 (2.0)
YAP-S physical activity (score)	Median (IQR)	2.6 (0.9)
YAP-S sedentary behaviors (score)	Median (IQR)	2.6 (0.8)
BMI (kg/m^2^)	Median (IQR)	21.7 (5.8)
BMI (z-score)	Median (IQR)	0.0 (1.9)
Overall sleep duration (minutes)	Median (IQR)	501.4 (72.9)
Energy intake (kcal)	Median (IQR)	2601.4 (1497.3)
Bedtime problems	No (%)	473 (75.9)
	Yes (%)	150 (24.1)
Excessive daytime sleepiness	No (%)	415 (66.6)
	Yes (%)	208 (33.4)
Awakenings during the night	No (%)	521 (83.6)
	Yes (%)	102 (16.4)
Problems with sleep regularity and duration	No (%)	434 (69.7)
	Yes (%)	189 (30.3)
Snoring	No (%)	585 (93.9)
	Yes (%)	38 (6.1)
Any sleep-related problem	No (%)	256 (41.1)
	Yes (%)	367 (58.9)
AAQ-II (score)	Median (IQR)	22.0 (17.0)

AAQ-II, Acceptance and Action Questionnaire-II; BMI, body mass index; FAS-III, Family Affluence Scale-III; IQR, interquartile range; YAP-S, Spanish Youth Activity Profile.

**Table 2:** Adjusted odds ratios for the association between AAQ-II score (per one point) and sleep-related problems.

Sleep Outcome	OR	95% CI Lower	95% CI Upper	*p*-Value
Bedtime problems	1.06	1.04	1.08	<0.001
Excessive daytime sleepiness	1.05	1.04	1.07	<0.001
Nocturnal awakenings	1.06	1.04	1.09	<0.001
Problems with sleep regularity and duration	1.04	1.02	1.06	<0.001
Snoring	1.01	0.97	1.05	0.537
Any sleep-related problem	1.06	1.04	1.08	<0.001

CI, confidence interval; OR, odds ratio.

## Data Availability

The data presented in this study are available on request from the corresponding author. As the participants are minors, privacy and confidentiality must be respected.

## Supplementary Materials

The following supporting information can be downloaded at: https://www.mdpi.com/article/10.3390/healthcare14142183/s1, Table S1: Comparison of participants with and without AAQ-II data; Table S2: Sensitivity analyses for AAQ-II associations; Table S3: Variance inflation factors from primary adjusted models; Table S4: Model calibration and discrimination indices; Table S5: Generalized linear model examining the association of Acceptance and Action Questionnaire-II with bedtime problems (and covariates) among adolescents; Table S6: Generalized linear model examining the association of Acceptance and Action Questionnaire-II with excessive daytime sleepiness (and covariates) among adolescents; Table S7: Generalized linear model examining the association of Acceptance and Action Questionnaire-II with awakenings during the night (and covariates) among adolescents; Table S8: Generalized linear model examining the association of Acceptance and Action Questionnaire-II with problems with sleep regularity and duration (and covariates) among adolescents; Table S9: Generalized linear model examining the association of Acceptance and Action Questionnaire-II with snoring (and covariates) among adolescents; Table S10: Generalized linear model examining the association of Acceptance and Action Questionnaire-II with any sleep-related problem (and covariates) among adolescents.
