# Supplementary material for: Exploring the Association Between Psychological Inflexibility, Experiential Avoidance and Sleep-Related Problems Among Adolescents: The EHDLA Study
Source: Healthcare (Basel). 2026 Jul 20;14(14):2183. doi: 10.3390/healthcare14142183 (PMC13410315; doi:10.3390/healthcare14142183)
Supplement: Supplementary file 1 [file healthcare-14-02183-s001.zip › healthcare-4377376-supplementary.pdf]

Supplementary Materials

Table S1. Comparison of participants with and without AAQ-II data.

Variable	Included (n = 717)	Excluded (n = 661)	p-value
Age (years)	14.0 (13.0, 15.0)	15.0 (13.0, 17.0)	<0.001
Girls, n (%)	396 (55.2%)	302 (45.7%)	<0.001
FAS-III (score)	8.0 (7.0, 9.0)	8.0 (7.0, 10.0)	0.800
BMI (z-score)	0.0 (-0.9, 1.0)	-0.0 (-0.9, 1.1)	0.883
Overall sleep duration (minutes)	497.1 (455.4, 527.1)	488.6 (450.0, 522.9)	0.005
YAP-S physical activity (score)	2.6 (2.2, 3.1)	2.6 (2.1, 3.1)	0.860
YAP-S sedentary behaviors (score)	2.6 (2.2, 3.0)	2.6 (2.2, 3.0)	0.046
Energy intake (kcal/day)	2590.0 (1958.2, 3469.6)	2590.9 (1978.9, 3504.5)	0.905
Any sleep-related problem, n (%)	415 (57.9%)	234 (35.4%)	<0.001

Included=participants with available AAQ-II data (n=717); final analytic sample (n = 623). Excluded=participants without AAQ-II data (n=661). Continuous variables are median (Q1, Q3). Categorical variables are n (%). AAQ-II=Acceptance and Action Questionnaire-II. FAS-III=Family Affluence Scale-III. BMI=body mass index. YAP-S=Spanish Youth Activity Profile.

Table S2. Sensitivity analyses for AAQ-II associations.

Outcome	Sensitivity analysis	OR	95% LLCI	95% ULCI	p-value
Bedtime problems	Primary (complete case)	1.06	1.04	1.08	<0.001
Excessive daytime sleepiness		1.05	1.04	1.07	<0.001
Nocturnal awakenings		1.06	1.04	1.09	<0.001
Irregular sleep regularity/duration		1.04	1.02	1.06	<0.001
Snoring		1.01	0.97	1.05	0.537
Any sleep-related problem		1.06	1.04	1.08	<0.001
Bedtime problems	Without sleep duration	1.06	1.04	1.08	<0.001
Excessive daytime sleepiness		1.06	1.04	1.08	<0.001
Nocturnal awakenings		1.06	1.04	1.09	<0.001
Irregular sleep regularity/duration		1.04	1.03	1.06	<0.001
Snoring		1.00	0.97	1.04	0.893
Any sleep-related problem		1.06	1.04	1.08	<0.001
Bedtime problems	Adjusted for DASS-21 anxiety and depression	1.04	1.02	1.07	0.001
Excessive daytime sleepiness		1.02	1.00	1.05	0.065
Nocturnal awakenings		1.06	1.03	1.09	<0.001
Irregular sleep regularity/duration		1.04	1.02	1.06	<0.001
Snoring		1.02	0.97	1.07	0.414
Any sleep-related problem		1.05	1.03	1.07	<0.001
Any sleep-related problem	AAQ-II tertile vs Low (reference): Medium	1.79	1.18	2.72	0.006
Any sleep-related problem	AAQ-II tertile vs Low (reference): High	3.51	2.22	5.52	<0.001
Bedtime problems	Sex-stratified: Boys	1.07	1.03	1.12	<0.001
Excessive daytime sleepiness		1.05	1.02	1.09	0.001
Nocturnal awakenings		1.06	1.01	1.11	0.018
Irregular sleep regularity/duration		1.06	1.03	1.09	<0.001
Snoring		1.01	0.97	1.06	0.615
Any sleep-related problem		1.05	1.02	1.08	<0.001
Bedtime problems	Sex-stratified: Girls	1.06	1.03	1.08	<0.001
Excessive daytime sleepiness		1.06	1.03	1.08	<0.001
Nocturnal awakenings		1.06	1.04	1.09	<0.001
Irregular sleep regularity/duration		1.04	1.01	1.06	0.001
Snoring		0.99	0.91	1.07	0.781
Any sleep-related problem		1.07	1.05	1.10	<0.001

AAQ-II=Acceptance and Action Questionnaire-II. OR=odds ratio. SE=standard error. LLCI=lower limit of the 95% confidence interval. ULCI=upper limit of the 95% confidence interval. DASS-21=Depression Anxiety Stress Scales-21. Unless otherwise stated, sensitivity analyses correspond to the AAQ-II (per one point) association.

Table S3. Variance inflation factors from primary adjusted models.

Outcome	Predictor	VIF
Bedtime problems	AAQ-II (per one point)	1.07
	Sex	1.12
	Age (per one year)	1.14
	FAS-III (per one point)	1.00
	Energy intake (per 1000 kcal)	1.06
	YAP-S physical activity (per one point)	1.09
	YAP-S sedentary behaviors (per one point)	1.23
	BMI z-score (per one unit)	1.03
	Overall sleep duration (per one hour)	1.19
Excessive daytime sleepiness	AAQ-II (per one point)	1.07
	Sex	1.17
	Age (per one year)	1.14
	FAS-III (per one point)	1.01
	Energy intake (per 1000 kcal)	1.05
	YAP-S physical activity (per one point)	1.07
	YAP-S sedentary behaviors (per one point)	1.23
	BMI z-score (per one unit)	1.04
	Overall sleep duration (per one hour)	1.16
Nocturnal awakenings	AAQ-II (per one point)	1.08
	Sex	1.10
	Age (per one year)	1.15
	FAS-III (per one point)	1.01
	Energy intake (per 1000 kcal)	1.06
	YAP-S physical activity (per one point)	1.08
	YAP-S sedentary behaviors (per one point)	1.23
	BMI z-score (per one unit)	1.03
	Overall sleep duration (per one hour)	1.23
Irregular sleep regularity/duration	AAQ-II (per one point)	1.10
	Sex	1.13
	Age (per one year)	1.17
	FAS-III (per one point)	1.01
	Energy intake (per 1000 kcal)	1.05
	YAP-S physical activity (per one point)	1.09
	YAP-S sedentary behaviors (per one point)	1.21
	BMI z-score (per one unit)	1.03
	Overall sleep duration (per one hour)	1.22
Snoring	AAQ-II (per one point)	1.14
	Sex	1.19

	Age (per one year)	1.13
	FAS-III (per one point)	1.05
	Energy intake (per 1000 kcal)	1.05
	YAP-S physical activity (per one point)	1.13
	YAP-S sedentary behaviors (per one point)	1.21
	BMI z-score (per one unit)	1.03
	Overall sleep duration (per one hour)	1.25
Any sleep-related problem	AAQ-II (per one point)	1.08
	Sex	1.16
	Age (per one year)	1.18
	FAS-III (per one point)	1.01
	Energy intake (per 1000 kcal)	1.03
	YAP-S physical activity (per one point)	1.10
	YAP-S sedentary behaviors (per one point)	1.18
	BMI z-score (per one unit)	1.03
	Overall sleep duration (per one hour)	1.18

VIF=variance inflation factor. AAQ-II=Acceptance and Action Questionnaire-II. FAS-III=Family Affluence Scale-III. BMI=body mass index. YAP-S=Spanish Youth Activity Profile.

Table S4. Model calibration and discrimination indices.

Outcome	AUC	HL chi-square	HL p-value
Bedtime problems	0.74	5.212	0.735
Excessive daytime sleepiness	0.76	14.747	0.064
Nocturnal awakenings	0.75	6.014	0.646
Irregular sleep regularity/duration	0.69	12.994	0.112
Snoring	0.70	28.727	<0.001
Any sleep-related problem	0.73	3.821	0.873

AUC=area under the receiver operating characteristic curve. HL=Hosmer–Lemeshow goodness-of-fit test. Values reflect discrimination and calibration of primary adjusted binomial generalized linear models for each sleep outcome.

Table S5. Generalized linear model examining the association of Acceptance and Action Questionnaire-II with bedtime problems (and covariates) among adolescents.

Predictor	OR	95% LLCI	95% ULCI	p-value
AAQ-II (per one point)	1.06	1.04	1.08	<0.001
Age (per one year)	0.93	0.80	1.07	0.300
Boys	Ref.			
Girls	1.58	0.99	2.52	0.056
FAS-III (per one point)	0.93	0.84	1.03	0.146
Energy intake (per 1000 kcal)	1.00	1.00	1.00	0.351
YAP-S physical activity (per one point)	0.82	0.59	1.15	0.247
YAP-S sedentary behaviors (per one point)	0.89	0.62	1.28	0.528
BMI z-score (per one unit)	1.16	0.99	1.36	0.058
Overall sleep duration (per one hour)	0.65	0.51	0.82	<0.001

AAQ-II=Acceptance and Action Questionnaire-II. FAS-III=Family Affluence Scale-III. BMI=body mass index. LLCI=lower limit of the 95% confidence interval. OR=odds ratio. Ref.=reference category. ULCI=upper limit of the 95% confidence interval. YAP-S=Spanish Youth Activity Profile. Models were adjusted for age, sex, socioeconomic status, energy intake, physical activity, sedentary behavior, BMI z-score, and sleep duration.

Table S6. Generalized linear model examining the association of Acceptance and Action Questionnaire-II with excessive daytime sleepiness (and covariates) among adolescents.

**Predictor**	**OR**	**95% LLCI**	**95% ULCI**	**p-value**
AAQ-II (per one point)	1.05	1.04	1.07	<0.001
Age (per one year)	1.08	0.95	1.23	0.241
Boys	Ref.			
Girls	2.29	1.49	3.52	<0.001
FAS-III (per one point)	0.95	0.86	1.04	0.236
Energy intake (per 1000 kcal)	1.00	1.00	1.00	0.499
YAP-S physical activity (per one point)	0.75	0.56	1.02	0.065
YAP-S sedentary behaviors (per one point)	1.99	1.41	2.79	<0.001
BMI z-score (per one unit)	0.98	0.85	1.13	0.761
Overall sleep duration (per one hour)	0.81	0.65	1.02	0.071

AAQ-II=Acceptance and Action Questionnaire-II. FAS-III=Family Affluence Scale-III. BMI=body mass index. LLCI=lower limit of the 95% confidence interval. OR=odds ratio. Ref.=reference category. ULCI=upper limit of the 95% confidence interval. YAP-S=Spanish Youth Activity Profile. Models were adjusted for age, sex, socioeconomic status, energy intake, physical activity, sedentary behavior, BMI z-score, and sleep duration.

Table S7. Generalized linear model examining the association of Acceptance and Action Questionnaire-II with awakenings during the night (and covariates) among adolescents.

**Predictor**	**OR**	**95% LLCI**	**95% ULCI**	**p-value**
AAQ-II (per one point)	1.06	1.04	1.09	<0.001
Age (per one year)	0.83	0.70	0.99	0.039
Boys	Ref.			
Girls	1.81	1.03	3.17	0.039
FAS-III (per one point)	0.97	0.86	1.09	0.623
Energy intake (per 1000 kcal)	1.00	1.00	1.00	0.011
YAP-S physical activity (per one point)	0.84	0.57	1.23	0.370
YAP-S sedentary behaviors (per one point)	1.02	0.68	1.54	0.924
BMI z-score (per one unit)	1.15	0.96	1.38	0.130
Overall sleep duration (per one hour)	0.91	0.69	1.19	0.478

AAQ-II=Acceptance and Action Questionnaire-II. FAS-III=Family Affluence Scale-III. BMI=body mass index. LLCI=lower limit of the 95% confidence interval. OR=odds ratio. Ref.=reference category. ULCI=upper limit of the 95% confidence interval. YAP-S=Spanish Youth Activity Profile. Models were adjusted for age, sex, socioeconomic status, energy intake, physical activity, sedentary behavior, BMI z-score, and sleep duration.

Table S8. Generalized linear model examining the association of Acceptance and Action Questionnaire-II with problems with sleep regularity and duration (and covariates) among adolescents.

Predictor	OR	95% LLCI	95% ULCI	p-value
AAQ-II (per one point)	1.04	1.02	1.06	<0.001
Age (per one year)	0.85	0.75	0.97	0.017
Boys	Ref.			
Girls	1.81	1.20	2.72	0.005
FAS-III (per one point)	0.93	0.85	1.01	0.093
Energy intake (per 1000 kcal)	1.00	1.00	1.00	0.205
YAP-S physical activity (per one point)	1.08	0.81	1.44	0.617
YAP-S sedentary behaviors (per one point)	0.96	0.70	1.33	0.820
BMI z-score (per one unit)	1.04	0.91	1.19	0.587
Overall sleep duration (per one hour)	0.77	0.62	0.96	0.020

AAQ-II=Acceptance and Action Questionnaire-II. FAS-III=Family Affluence Scale-III. BMI=body mass index. LLCI=lower limit of the 95% confidence interval. OR=odds ratio. Ref.=reference category. ULCI=upper limit of the 95% confidence interval. YAP-S=Spanish Youth Activity Profile. Models were adjusted for age, sex, socioeconomic status, energy intake, physical activity, sedentary behavior, BMI z-score, and sleep duration.

Table S9. Generalized linear model examining the association of Acceptance and Action Questionnaire-II with snoring (and covariates) among adolescents.

**Predictor**	**OR**	**95% LLCI**	**95% ULCI**	**p-value**
AAQ-II (per one point)	1.01	0.97	1.05	0.537
Age (per one year)	1.07	0.82	1.38	0.625
Boys	Ref.			
Girls	0.20	0.08	0.51	<0.001
FAS-III (per one point)	1.07	0.88	1.30	0.497
Energy intake (per 1000 kcal)	1.00	1.00	1.00	0.414
YAP-S physical activity (per one point)	0.54	0.29	1.01	0.055
YAP-S sedentary behaviors (per one point)	0.90	0.46	1.75	0.754
BMI z-score (per one unit)	1.20	0.92	1.55	0.178
Overall sleep duration (per one hour)	1.51	0.89	2.54	0.123

AAQ-II=Acceptance and Action Questionnaire-II. FAS-III=Family Affluence Scale-III. BMI=body mass index. LLCI=lower limit of the 95% confidence interval. OR=odds ratio. Ref.=reference category. ULCI=upper limit of the 95% confidence interval. YAP-S=Spanish Youth Activity Profile. Models were adjusted for age, sex, socioeconomic status, energy intake, physical activity, sedentary behavior, BMI z-score, and sleep duration.

Table S10. Generalized linear model examining the association of Acceptance and Action Questionnaire-II with any sleep-related problem (and covariates) among adolescents.

Predictor	OR	95% LLCI	95% ULCI	p-value
AAQ-II (per one point)	1.06	1.04	1.08	<0.001
Age (per one year)	0.90	0.79	1.01	0.078
Boys	Ref.			
Girls	1.77	1.21	2.58	0.003
FAS-III (per one point)	0.95	0.87	1.04	0.280
Energy intake (per 1000 kcal)	1.00	1.00	1.00	0.045
YAP-S physical activity (per one point)	0.79	0.60	1.03	0.086
YAP-S sedentary behaviors (per one point)	1.38	1.01	1.90	0.046
BMI z-score (per one unit)	1.07	0.94	1.22	0.281
Overall sleep duration (per one hour)	0.86	0.69	1.07	0.181

AAQ-II=Acceptance and Action Questionnaire-II. FAS-III=Family Affluence Scale-III. BMI=body mass index. LLCI=lower limit of the 95% confidence interval. OR=odds ratio. Ref.=reference category. ULCI=upper limit of the 95% confidence interval. YAP-S=Spanish Youth Activity Profile. Models were adjusted for age, sex, socioeconomic status, energy intake, physical activity, sedentary behavior, BMI z-score, and sleep duration.
